# Transition Metal Carbides as Supports for Catalytic Metal Particles: Recent Progress and Opportunities

**DOI:** 10.1021/acs.jpclett.3c03214

**Published:** 2024-03-21

**Authors:** Hector Prats, Michail Stamatakis

**Affiliations:** † Department of Chemical Engineering, University College London, Roberts Building Torrington Place, London WC1E 7JE, U.K.; ‡ Department of Chemistry, Inorganic Chemistry Lab, University of Oxford, Oxford OX1 3QR, U.K.

## Abstract

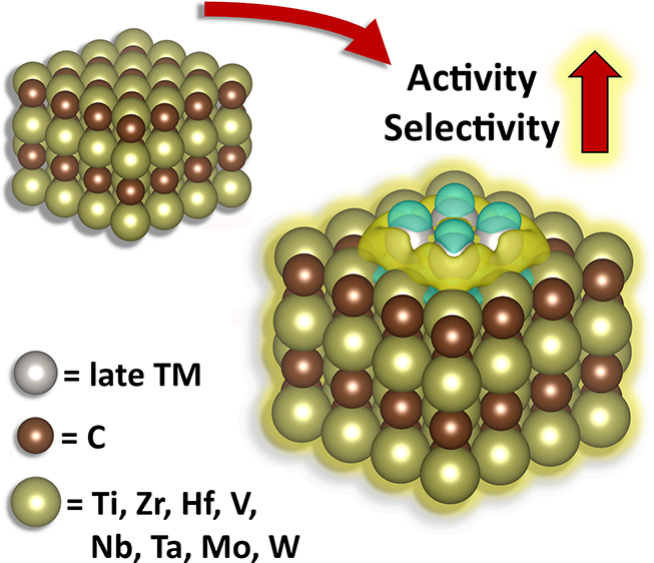

Transition metal carbides (TMCs) constitute excellent alternatives to traditional oxide-based supports for small metal particles, leading to strong metal–support interactions, which drastically modify the catalytic properties of the supported metal atoms. Moreover, they possess extremely high melting points and good resistance to carbon deposition and sulfur poisoning, and the catalytic activities of some TMCs *per se* have been shown to be similar to those of Pt-group metals for a considerable number of reactions. Therefore, the use of TMCs as supports can give rise to bifunctional catalysts with multiple active sites. However, at present, only TiC and Mo_*x*_C have been tested experimentally as supports for metal particles, and it is largely unclear which combinations may best catalyze which chemical reactions. In this Perspective, we review the most significant works on the use of TMCs as supports for catalytic applications, assess the current status of the field, and identify key advances being made and challenges, with an eye to the future.

Transition metal carbides (TMCs) are obtained by incorporating C atoms into the lattice of transition metals of groups 3–10, although only those of groups 4–6 have been extensively studied by both theory and experiment because they are thermodynamically more stable (Figure 1). In general, TMCs possess unique physical and chemical properties due to a bonding that involves covalent, ionic, and metallic contributions,^1,2^ which has led to their use in various commercial applications, such as cutting tools^1^ or hard-coating materials.^3^ Due to the incorporation of C atoms at the interstitial sites of the transition metal (TM) lattice, TMCs possess much higher density of states near the Fermi level, resulting in a noble-metal-like electron configuration and catalytic behavior. Ever since the landmark paper by Levy and Boudart in 1973 regarding the Pt-like properties of WC as a catalyst *per se*,^4^ the catalytic properties of TMCs have been the subject of many experimental studies, often accompanied by theoretical investigations, showing a strong similarity to those of more expensive noble metals^5^ for a variety of reactions such as desulphurization,^6^ methane reforming,^7^ or the (reverse) water–gas shift (WGS) reaction.^8,9^

**Figure 1:**
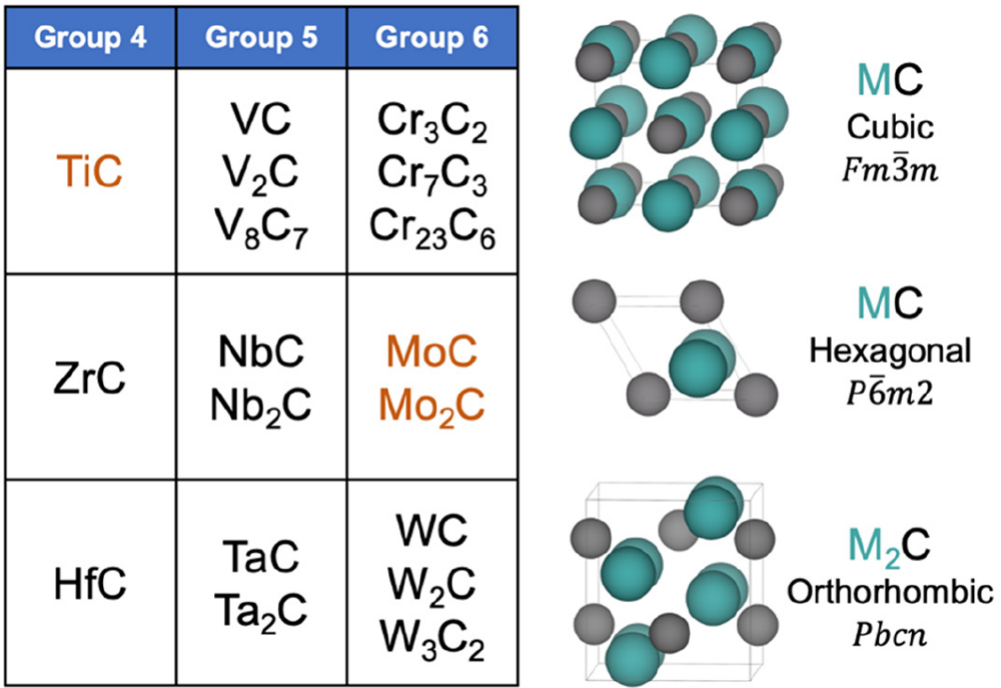
Transition metal carbides from groups 4–6^18^ and primitive cell of the cubic, hexagonal, and orthorhombic phases. Carbides in brown are the only ones that have been studied extensively as supports experimentally.

Apart from the use of TMCs *per se* as catalytic materials, a new line of research originated in the late 2000s on the use of TMCs as supports for small TM particles,^10^ namely, TM/TMC or TM@TMC, demonstrating higher activities and selectivities than those obtained when using oxide supports.^11^ The enhanced activity is attributed to a strong polarization of the electron density of the metal particle when supported to a TMC^12^ and the fact that, unlike traditional support materials that are inert, the use of TMCs as active supports leads to bifunctional catalysts with multiple active sites allowing for cooperative effects.^13,14^ Also, pure TMCs tend to be oxidized and deactivated in oxidative reaction environments at high temperatures leading to the formation of surface oxycarbides. However, supported metal clusters can facilitate the turnover of oxygen-containing species, such as OH or O on the TMC, preventing the deactivation of the catalyst. Since the late 2000s, a significant number of works have reported the outstanding catalytic performance of TMC-supported metal particles for a variety of reactions, such as the WGS reaction,^15^ CO_2_ hydrogenation,^16^ and low-temperature CH_4_ activation,^17^ among others. To date, however, only a very small subset of TM/TMC catalysts has been tested experimentally. This Perspective summarizes both experimental and theoretical results on the use of TMCs as supports for metal particles; identifies the key factors defining activity, selectivity, and stability; and illustrates important trends for the rational design of novel TM/TMC catalysts, with the aim to shed light on future development of these materials.

## Everything Started with Au/TiC

**Figure 2:**
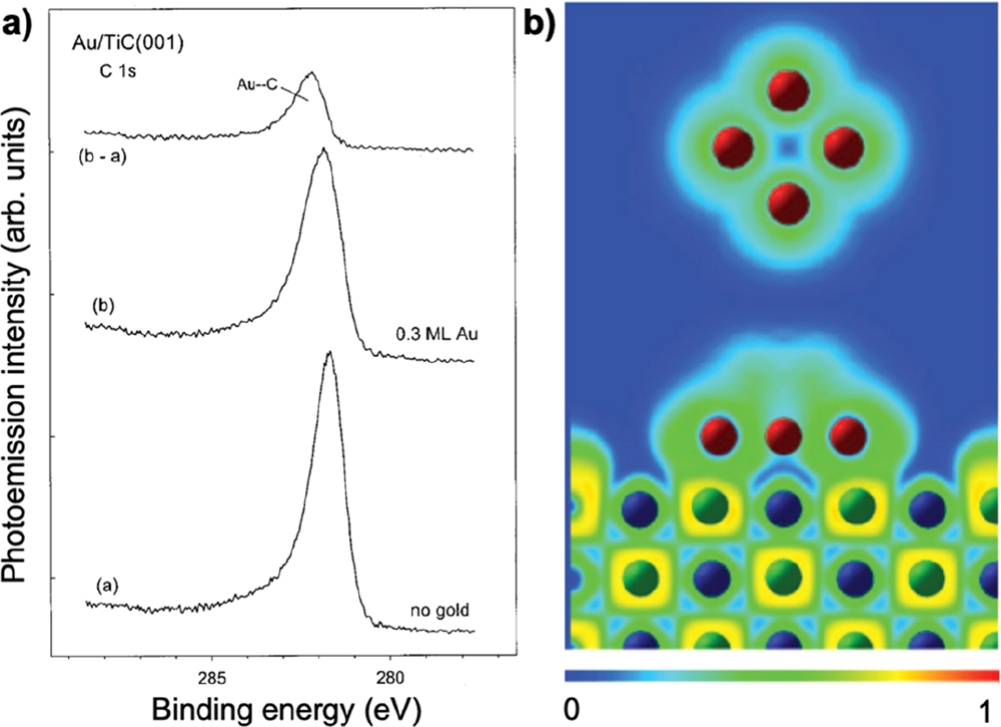
(a) C 1s photoemission data for clean TiC(001) and Au/TiC(001) system obtained after the deposition of 0.3 ML of Au at 300 K. A photon energy of 380 eV was used to excite the electrons. Before performing the (“b” – “a”) subtraction, the peak intensity of spectrum “a” was renormalized. (Adapted with permission from ref (12). Copyright 2007 American Institute of Physics.) (b) ELF map for Au_4_ adsorption on TiC(001). At the top, the corresponding result for the isolated Au_4_ cluster is shown. The probability of finding one electron varies from 0 (blue color) to 1 (red color). (Adapted with permission from ref (12). Copyright 2007 American Institute of Physics.)

The first reports on the use of TMCs as supports date back to the late 2000s. Motivated by the great potential to use TMCs as catalytic supports in industrial applications due to their hardness, high melting points, and corrosion resistance,^19,20^ Ono et al. studied the catalytic properties of dispersed Au nanoparticles on an ultrathin TiC film, showing the resulting material’s ability to catalyze the oxidation of CO at temperatures below 200 K.^10^ Temperature-programmed desorption (TPD) results indicated an enhancement of the catalytic activity with decreasing particle size and higher stability toward agglomeration for the system with the largest average interparticle distance.^21^ Scanning tunnelling spectroscopy (STM) experiments pointed to a transition from metallic to nonmetallic behavior with decreasing Au particle size.^22^ Based on these findings, Rodriguez et al. investigated the adsorption of Au on a well-defined TiC(001) surface using synchrotron-based high-resolution photoemission and density functional theory (DFT) calculations.^12^ A positive shift in the binding energy of the C 1s core level was observed after the deposition of Au, indicating the formation of Au–C bonds. DFT calculations corroborated the formation of these bonds and showed that, despite the very little ionic character thereof, there is a substantial polarization of electrons around Au that affects its chemical properties (Figure 2). In particular, the polarization of charge around gold facilitates bonding with electron-acceptor molecules such as CO, O_2_, C_2_H_4_, C_2_H_2_, or SO_2_ and produces systems with high catalytic activity.

Subsequent experiments showed that Au/TiC is extremely active for desulphurization (DeSO_*x*_) processes and is more efficient than either Au/MgO or Au/TiO_2_.^23^ Au/TiC can break both S–O bonds at a temperature as low as 150 K. Moreover, it was shown that the size of the Au particle has a drastic effect on the reactivity since the effects of the Au–TiC(001) interactions are significant only for small Au clusters. STM images pointed to a very high DeSO_*x*_ activity when Au particles are smaller than 1.5 nm, and a substantial decrease of activity was observed when the Au particle size exceeds 2 nm. DFT calculations corroborated that small Au clusters (4–13 atoms) are more active than the bigger ones. Motivated by these findings, Rodriguez et al. investigated the hydrodesulphurization of thiophene on Au/TiC.^24^ In spite of the very poor performance of TiC(001) or Au(111), Au/TiC(001) is more active than conventional Ni/MoS_*x*_ catalysts, and it was suggested that the substantial polarization of electron density around the Au clusters facilitates the dissociation of H_2_, providing the H atoms necessary for the DeSO_*x*_ of thiophene. Florez et al. studied in more detail the dissociation of H_2_ on Au/TiC by performing DFT calculations on a variety of cluster models, presenting theoretical evidence that small two-dimensional Au particles supported on TiC(001) are more efficient at dissociating H_2_ than when supported on oxides.^25^ These findings inspired further experiments in hydrogenation reactions on Au/TiC, and in the following years it was shown that indeed Au/TiC is more active for the CO_2_ hydrogenation to methanol than conventional Cu/ZnO catalysts.^26,27^ Other joint experimental and theoretical studies also reported the exceptional catalytic performance of Au/TiC toward low-temperature O_2_ dissociation^28^ and low-temperature WGS reaction.^29^ In all cases, small 2D particles were shown to be the most reactive.

## TiC-Supported Catalysts

In view of the above-mentioned results for Au/TiC, the following question has arisen: does this substantial polarization of the electron density also occur for metal particles different than Au or for TMC supports different than TiC? In this section, we will focus on TiC-supported metal particles, and other TMC supports will be discussed in the next sections. To our knowledge, the first work on other metal particles supported on TiC was performed by Gómez et al., who adopted DFT to study the reactivity of Pd_4_, Pt_4_, Cu_4_, Ag_4_, and Au_4_ clusters supported on TiC(001) toward H_2_ dissociation.^30^ The results from these DFT calculations indicated that while H_2_ dissociation on Pd_4_/TiC and Pt_4_/TiC is more difficult than on the corresponding extended TM (111) and (001) surfaces, the opposite trend is observed on Cu_4_/TiC, Ag_4_/TiC, and Au_4_/TiC, with Cu_4_/TiC and Au_4_/TiC exhibiting the lowest dissociation energy barriers. Motivated by those findings, Feria et al. studied the DeSO_*x*_ activity on Cu/TiC using experiments and theory.^31^ Both experiments and DFT calculations indicate that Cu/TiC is even more active than Au/TiC for DeSO_*x*_, and is also able to catalyze the Claus reaction (SO_2_ + 2H_2_S → 3S + 2H_2_O) and the reduction of sulfur dioxide by CO (SO_2_ + 2CO → 2CO_2_ + S) under high-vacuum conditions.

**Figure 3:**
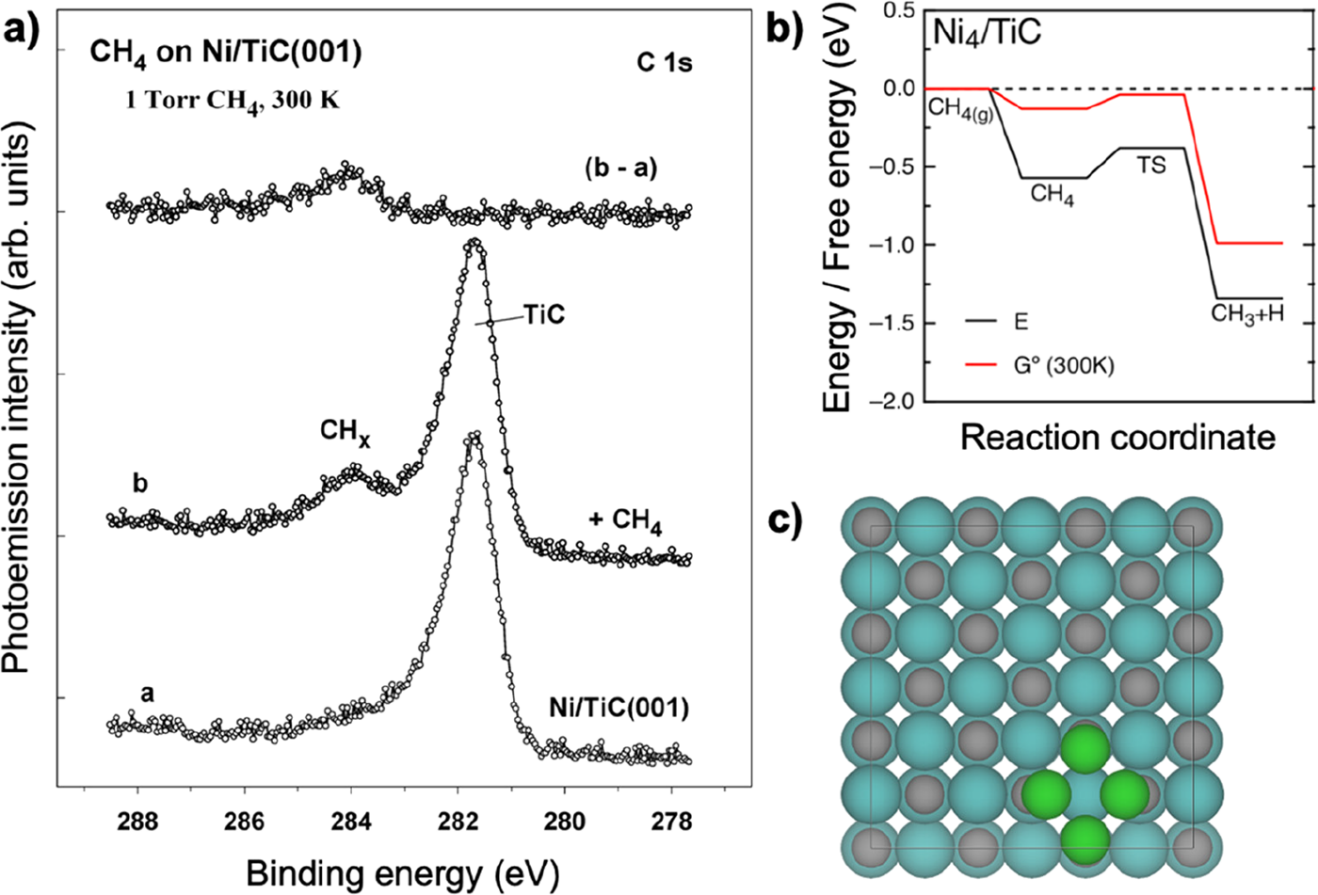
(a) C 1s ZPS spectra collected before and after dosing methane to Ni/TiC(001) at 300 K. The dosage of methane was 1 Torr for 5 min. The coverage of Ni on the TiC(001) substrate was 0.2 ML. (Adapted with permission from ref (17). Copyright 2019 American Chemical Society.) (b) Potential energy profile (black) and free energy profile at 300 K and 1 atm of CH_4_ (red) for CH_4_ adsorption and dissociation on a model of Ni_4_/TiC(001). (Adapted with permission from ref (17). Copyright 2019 American Chemical Society.) (c) Computational model of Ni_4_/TiC(001) used in ref (17).

Shortly after, the CO_2_ hydrogenation activity of Cu/TiC and Ni/TiC compared to Au/TiC was studied experimentally by Vidal et al.^26^ and Rodriguez et al.^27^ The major product over these catalysts is CO, which results from the reverse WGS reaction (CO_2_ + H_2_ → CO + H_2_O). In Au/TiC and Cu/TiC, a substantial amount of methanol was also produced, but no methane was detected. In contrast, Ni/TiC produces a mixture of CO, methanol, and methane. The experiments showed that Cu/TiC and Ni/TiC outperform Au/TiC in terms of CO produced in all range of temperatures considered, and the TOFs for methanol production on Cu/TiC is between 3 and 8 times higher than on Au/TiC and Ni/TiC. Moreover, the catalytic activity of these TMC-supported metals can be orders of magnitude higher than that on the corresponding extended TM surfaces.^27^ As shown in a DFT study by Lozano-Reis et al., the enhanced activity of Ni/TiC is also attributed to the polarization that TiC inflicts on the electronic density of the supported Ni particles,^32^ which greatly reduces the dissociation energy barriers for CO_2_ and H_2_.^33^ Finally, in a recent joint experimental and computational study, Prats et al. showed that Ni/TiC is able to activate methane at room temperature (Figure 3),^17^ which is a major challenge due to the high stability of the C–H bond (i.e., 4.5 eV dissociation energy in vacuum).

## Mo_*x*_C-Supported Catalysts

**Figure 4:**
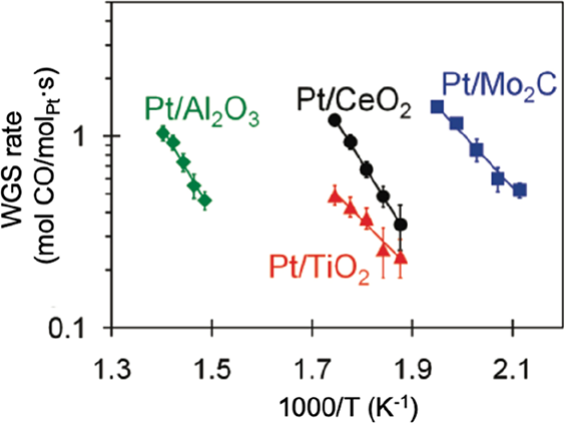
Arrhenius plots of the WGS reaction rates for 2.7% Pt/Al_2_O_3_, 5% Pt/CeO_2_, 2% Pt/TiO_2_, and 4% Pt/Mo_2_C catalysts. (Adapted with permission from ref (36). Copyright 2011 American Chemical Society.)

Apart from TiC, another TMC that has been extensively studied as a support for metal particles is Mo_*x*_C. Back in 2004, Griboval-Constant et al. reported that supporting Co or Ru onto Mo_2_C increases the activity toward Fischer–Tropsch synthesis and modifies the product distribution.^34^ In another study, Lewandowski et al. dispersed Pt on Mo_2_C and found the resulting material to be highly active for simultaneous hydrodenitrogenation and hydrodesulphurization reactions.^35^ However, in these studies, the metals were deposited onto passivated Mo_2_C powders. Therefore, the metal precursors interacted with an oxidized surface rather than the native carbide surface. The first investigation on metal clusters directly supported to Mo_2_C was done in 2011 by Schweitzer et al, who studied the WGS reaction on 2–4 nm Pt particles supported on unpassivated Mo_2_C.^36^ The reaction rate on this catalyst was higher than those for the most active oxide-supported Pt catalysts (e.g., Pt/CeO_2_ and Pt/TiO_2_) and the commercial Cu–Zn–Al catalyst (Figure 4). Experimental and computational results suggested that the reactivity occurs on the perimeter of the Pt particles and that the strong Pt–Mo_2_C interactions give rise to a raft-like morphology, which is advantageous due to its high surface area to volume ratio. In light of this work, Sabnis et al. studied the role of the admetal for promotion of the WGS activity by preparing supported Pt, Pd, Au, Ni, Cu, and Ag catalysts on Mo_2_C,^37^ although in this case, the carbide surface was passivated. The measured TOF was between 3 and 6 times higher compared to bare Mo_2_C with Pt, Au, Pd, and Ni particles, while no significant improvement was observed with the addition of Cu and Ag.

A significant breakthrough in the use of molybdenum carbide as a support was made by Xu et al. in 2015,^16^ who reported Cu/Mo_2_C, Ni/Mo_2_C, and Co/Mo_2_C as highly active catalysts for CO_2_ hydrogenation. Large variations in the selectivity were observed depending on the nature of the supported metal, with Cu, Ni, and Co exhibiting significant selectivity toward methanol, methane, and hydrocarbons, respectively. Subsequent experiments on clean Mo_2_C showed that it is selective toward methane,^38^ but there is a shift in selectivity from methane to methanol after Cu deposition due to a new pathway for methanol production at the Cu–Mo_2_C interface. In a joint experimental and computational study published in the following year, Posada-Pérez et al.^39^ compared the catalytic performance of Au particles supported on MoC and Mo_2_C for the hydrogenation of CO_2_ and demonstrated that the metal/C ratio is a key determining factor of activity, selectivity, and stability. On clean MoC, only CO and methanol were detected as products. In contrast, on Mo_2_C there was production of a large amount of methane in addition to CO and methanol. The addition of Au clusters enhances the rates of formation of all products in both carbides, but while the rates of CO formation on Au/MoC and Au/Mo_2_C are comparable, there is no methane formation on Au/MoC. This increase in selectivity was accompanied by an increase in stability, as shown by XPS measurements after reaction. On the MoC substrate, a minor amount of oxygen (∼0.1 ML) was found, and the O coverage did not increase with time. However, the amount of O present on Mo_2_C after reaction was large (>0.4 ML) and increased with time, inducing a drop in catalytic activity due to O poisoning. Note that TMCs can be oxidized at high temperatures in a process that removes C atoms from the surface and ultimately leads to the formation of *oxycarbides*,^40^ thereby drastically modifying their chemical properties. In the same study, the authors also compared the activity of Au/MoC with Cu/MoC, finding that the CO rate on the latter is about 5 times higher compared to Au/MoC. Subsequent DFT calculations suggested that Cu/MoC works as a bifunctional catalyst, where Cu clusters dissociate CO_2_ and MoC catalyzes the main hydrogenation steps.

The effect of the metal/C ratio was also studied for the WGS reaction activity on Pt^41^ and Au^42^ clusters supported on MoC and Mo_2_C. Again, MoC-supported particles showed higher stability and good selectivity, while Mo_2_C-supported particles suffer from deactivation due to oxycarbide formation, as well as lower selectivity due to methane formation. The better stability of MoC-supported particles is attributed to MoC having lower affinity for water and binding OH and O species more weakly than Mo_2_C. In fact, atomic-layered Au clusters on MoC were shown to be very active and stable for the low-temperature WGS reaction at temperatures up to 473 K.^15^

MoC has also been shown to be an excellent support for single-atom catalysts (SACs), as described in a recent review article.^43^ For instance, atomically dispersed Pt atoms on MoC (Pt_1_/MoC) are highly active and stable for the aqueous-phase reforming of methanol (APRM).^44^ While MoC provides active sites for water dissociation, electron-deficient supported Pt atoms favor the adsorption and activation of methanol. Recently, Zhang et al. showed that crowding the MoC surface with Pt_1_ and Pt_*n*_ species can prevent oxidation of the support that would cause catalyst deactivation,^45^ as seen with Au/MoC.^15^ Specifically, supported Pt species effectively prompt the turnover of oxygen species on the adjacent MoC sites with CO adsorbed on Pt. Atomically dispersed Ni_1_/MoC has also been reported to be an outstanding catalyst for APRM.^46^ X-ray absorption fine structure (EXAFS) and DFT calculations indicate that Ni_1_–C_*x*_ motifs are formed, which can effectively stabilize the isolated Ni_1_ sites over the MoC substrate, thereby maximizing active site density and delivering high structural stability. Finally, a recent work by Ge et al. reported the synthesis of a highly dispersed CoNi bimetallic catalyst supported on MoC for the efficient hydrogen production from the hydrolysis of ammonia borane.^47^ The metal-normalized activity of this catalyst surpasses all the noble metal-free catalysts ever reported and is four times higher than that of the commercial Pt/C catalyst. The improved catalytic performance is due to the synergistic effect between the nearly atomically dispersed Co and Ni atoms.

## Other TMC Supports

TiC and Mo_*x*_C have dominated the study of TMCs as catalytically active supports for metal particles, and only a few theoretical works have considered other TMCs. To our knowledge, the first work to do so was performed by Florez et al. in 2009,^48^ who studied the adsorption and diffusion of Au atoms on the (001) surface of Ti, Zr, Hf, V, Nb, Ta, and Mo carbides. By employing DFT calculations, the authors showed that the adsorption energy of Au on these surfaces is moderately large, making diffusion possible before desorption could occur. In general, there is noticeable charge transfer from the TMC to the Au atom, especially for the case of ZrC and TaC, suggesting that Au/ZrC and Au/TaC could outperform Au/TiC in catalytic applications. Subsequent theoretical calculations by Gómez et al. studied the interaction of atoms of groups 9–11 with the (001) surface of TiC, ZrC, VC, and δ-MoC,^49^ showing that the binding to the TMC is especially strong for atoms of groups 9 and 10, and on average the strongest charge transfer is observed for the case of atoms supported on ZrC and TiC.

As far as we are aware, there has been no experimental study on the use of other carbide supports apart from TiC and Mo_*x*_C so far, despite the previously mentioned results. However, a recent DFT-based high-throughput screening study on the electronic properties of 7 nanoclusters (Rh, Pd, Pt, Au, Co, Ni, and Cu) on 11 stable support surfaces of TMCs with 1:1 stoichiometry (TiC, ZrC, HfC, VC, NbC, TaC, MoC, and WC) unravelled several interesting trends and properties.^50^ Regarding the choice of systems considered in this study, note that for all the above-mentioned carbides the cubic phase is stable, with the (001) surface being the lowest-energy one, but for MoC and WC a hexagonal phase is also stable, with the metal- or C-terminated (0001) facet being the lowest-energy one. Thus, for hexagonal MoC both terminations were considered, while for hexagonal WC only the W-termination was studied, as the C-termination is much less stable.

**Figure 5:**
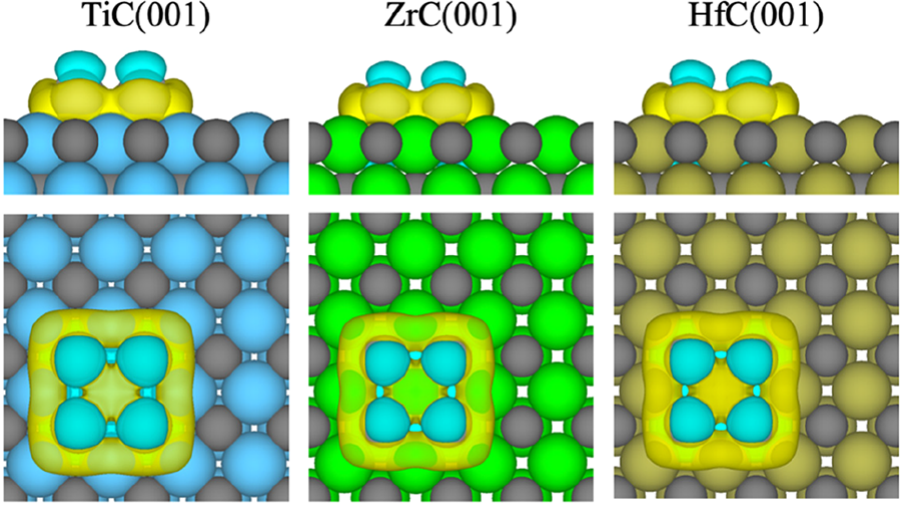
Charge density difference plots for Pd clusters supported on selected TMCs. The isosurface level is taken as 0.001 *e*·bohr^–3^. Yellow regions denote accumulation of charge density, while blue regions denote charge density depletion. (Replotted from the data calculated in ref (50).)

The DFT calculations of this study showed that the strong polarization of the electron density in the supported metal particle, induced by the TMC, is not an exclusive phenomenon of Au/TiC but rather a general property of TM/TMCs.^50^ In fact, the strongest polarization of the electron density is not displayed by Au clusters but by Pt, Pd, and Rh clusters. This result can be explained by the higher polarizability of larger atoms, which have more loosely held electrons and more diffuse orbitals. Interestingly, most systems show an accumulation of charge density on the interface and a depletion on top of the cluster (Figure 5), which should facilitate bonding of electron-acceptor molecules (e.g., O_2_, CO_2_, SO_2_, etc.) to the interface and bonding of electron-donor molecules (e.g., H_2_, CO, NH_3_, etc.) on top of the cluster atoms. Finally, the calculations also indicated that it is possible to manipulate the charge state (i.e., partially oxidized or partially reduced) of the supported cluster by choosing TMC metal and cluster metal atoms with custom electronegativities, and by doing so, it is possible to facilitate or block the bonding of certain molecules.

In general, for any adsorbed species there are some TM/TMC combinations that bind it more strongly than the corresponding extended TM and TMC surfaces alone.^51^ However, the binding strength of the adsorbate ultimately depends on the specific combination of TM and TMC used, and it is not possible to accurately predict it from the binding strengths to the clean TM and TMC surfaces due to the special polarization of the electron density of such supported metal particles (Figure 2b in ref (51)). Unfortunately, descriptor-driven screening of TM/TMCs is quite challenging, since the simple linear scaling relations that exist between the adsorption energies of chemically similar adsorbates in extended TMs (e.g., CH_*x*_ vs C or OH_*x*_ vs O) are not obeyed by TM/TMCs or even extended TMCs surfaces,^51^ as a consequence of the higher diversity of adsorption sites and the more ionic nature of the interactions.

**Figure 6:**
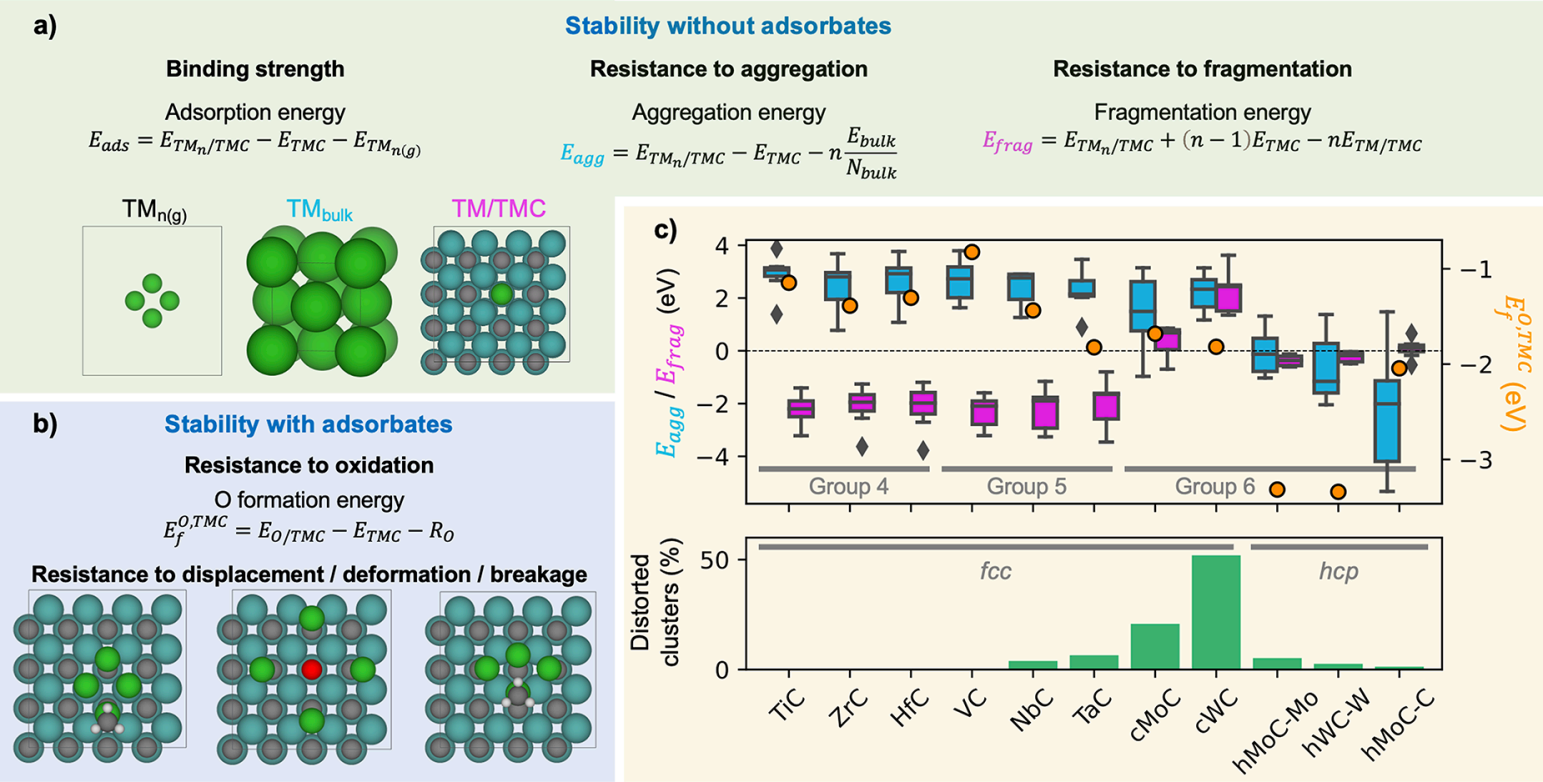
(a) Computed properties adopted in ref (52) to predict the stability of the clean supported clusters (without adsorbates). , *E*_TMC_, *E*_TM_*n*(g)__, *E*_bulk_, and *E*_TM/TMC_ are the total DFT energies of the TMC-supported cluster, the clean TMC, the TM cluster in the gas-phase, the bulk TM, and a single TM atom supported on the TMC, respectively, while *N*_bulk_ is the number of atoms in the bulk unit cell. (b) Definition of *E*_f_^O,TMC^ and examples of displaced (CH_3_ on Au_4_/TaC), deformed (CH_3_ on Au_4_/NbC), and broken clusters (CO on Pt_4_/cWC). In the equation, *E*_O/TMC_ and *E*_TMC_ are the total DFT energies of a single oxygen adatom on TMC and of the bare TMC surface, while *R*_O_ is the reference energy for the O atom in vacuum, calculated with respect to H_2_, CH_4_, and CO_2_ as 1/2*E*_CO_2(g)__ – 1/2*E*_CH_4(g)__ + *E*_H_2(g)__. (c) (Top panel) Distribution of *E*_agg_ (cyan box plots), *E*_frag_ (magenta box plots), and *E*_f,O_ (orange dots) for metal clusters supported on 11 TMC facets calculated in ref (52). Note that more negative *E*_agg_ and *E*_frag_ mean higher stability, while more negative *E*_f_^O,TMC^ means stronger bonding to O and therefore lower resistance against oxidation. The box limits correspond to the interquartile range (IQR), formed between the first and third quartiles (Q1 and Q3), and the whiskers (black lines) extend to Q1 – 1.5·IQR and Q3 + 1.5·IQR. Outliers are shown as black rhombi. (Bottom panel). Percentage of TM clusters that are distorted (i.e., TM–TM bond contraction or elongation higher than 35% or cluster displacement higher than 0.5 Å per atom after the adsorption of an adsorbed species). The cubic *fcc* and hexagonal *hcp* phases for MoC and WC are referred to as cTMC and hTMC, respectively. (Replotted from the data calculated in ref (52).)

The thermodynamic stability of the supported clusters can be evaluated from DFT calculations by computing several properties. For instance, the binding strength of the supported cluster to the carbide and its resistance against aggregation and fragmentation can be evaluated by computing the adsorption energy (*E*_ads_), aggregation energy (*E*_agg_), and fragmentation energy (*E*_frag_), respectively, as illustrated in Figure 6a. Within these definitions, a more negative *E*_ads_ means stronger binding, and more negative *E*_agg_ and *E*_frag_ mean higher stability. Some important trends in stability were elucidated in another recent DFT-based high-throughput screening study on the same systems (i.e., the 77 TM/TMC combinations mentioned above).^52^ For instance, the nature of the TMC is predicted to be very important in terms of its stability against aggregation or fragmentation (Figure 6c). Clusters supported on group 4 (Ti, Zr, Hf) and 5 (V, Nb, Ta) TMCs are resistant against fragmentation but weak against aggregation. For the case of group 6 (Mo and W) TMCs, the stability against fragmentation and aggregation is strong on hexagonal MoC and WC but weak on cubic MoC and WC.

Another important consideration is the cluster stability in the presence of adsorbates. Small clusters are generally labile, and thus, the bonds between the cluster atoms can be extended or contracted to accommodate the different reactants. This lability was predicted to be critical for the low-temperature activation of CH_4_ on Ni/TiC,^17^ but the disadvantage is that the supported clusters may deform significantly, be displaced, or even break as a result of interacting with reaction intermediates (Figure 6b). By computing the interactions of all 77 TM/TMCs with an array of catalytically relevant molecules or molecular fragments, it was shown that the cluster stability strongly depends on the chosen TMC support, with clusters on TiC, ZrC, HfC, and VC being always very rigid and clusters on cubic MoC and WC being more likely to be displaced, deformed, or broken (Figure 6c). Regarding the nature of the cluster, the stability follows the trend group 9 (Co, Rh) > group 10 (Ni, Pd, Pt) > group 11 (Cu, Au). Most importantly, it was shown that *E*_frag_ and *E*_ads_ are excellent descriptors for cluster stability in the presence of adsorbates.

Finally, the resistance of TM/TMC catalysts against oxidation was evaluated from the formation energy of O species on the supported clusters () and the TMC support (*E*_f_^O,TMC^), which is a measure of their binding strength to O (the more negative, the stronger the binding).  mainly depends on the nature of the cluster atoms and follows the trend of O binding in extended TM surfaces (Au > Pt > Pd > Cu > Rh > Ni > Co). On TMCs, the strongest binding is found in hexagonal carbides (especially in the metal-termination), and for cubic carbides, *E*_f_^O,TMC^ follows the trend VC > TiC > HfC > ZrC > NbC > MoC > WC > TaC (Figure 6c). Therefore, Au/VC is predicted as the most resistant combination against oxidation.

**Figure 7:**
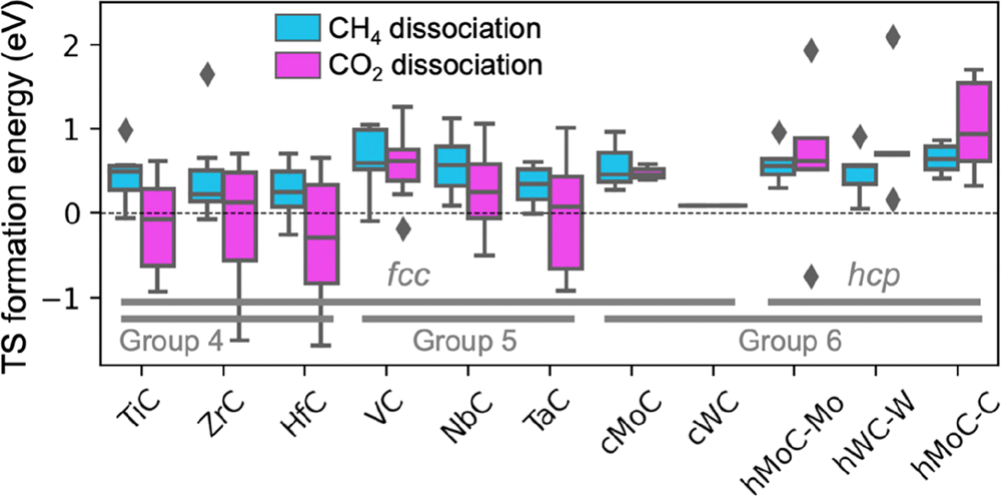
(a) Box plots showing the distribution of formation energies for the transition states of CH_4_ (cyan) and CO_2_ (magenta) dissociation on metal clusters supported on 11 TMC facets. Further information on the box plot limits, notation, and computational details and models can be found in the caption of Figure 6. (Replotted from the data calculated in ref (52).)

With regard to the reactivity of the supported clusters, the potential catalytic activity toward CH_4_ and CO_2_ conversion technologies was evaluated in the same study^52^ by computing the transition state formation energies for the first bond breaking of these molecules (i.e., energy of the CH_3_–H and CO-O transition state with respect to gas-phase CH_4_ and CO_2_, respectively). Many TM/TMC combinations can dissociate CO_2_ and CH_4_ with negligible energy barriers. Among the different TMs studied, Pt clusters have on average the lowest energy barriers. As for the TMC supports, the highest reactivity is predicted for metal clusters supported on TMCs made from group 4 elements (Ti, Zr, and Hf) or Ta (Figure 7). CO_2_ dissociation is, in general, easier than that of CH_4_ due to the ability of CO_2_ to accept electrons into its lowest unoccupied molecular orbital to form negatively charged bent species (CO_2_^∂-^), weakening the C–O bonds. By considering all stability and activity metrics, Pd/ZrC, Pt/ZrC, Pd/HfC, Pt/HfC, Ni/VC, Pd/VC, Ni/NbC, and Pd/NbC were identified as promising candidates with high stability and catalytic performance, all of them being new for experimental validation.

## Kinetic Modeling of TM/TMC Catalysts

DFT-derived free energy profiles can provide useful insights into the reaction mechanism at the molecular level, but in the case of bifunctional catalysts with multiple types of active sites, such as TM/TMCs, the number of elementary steps that may occur skyrockets, making the analysis of free energy profiles very complicated. Moreover, such profiles do not easily account for the effect of surface coverage, which would require updating the potential energy surface for each configuration of spectator species, which is impractical, and they neglect configurational entropy. Site blocking and lateral interactions with spectator species can play a key role in determining the catalytic activity,^14^ and thus, DFT calculations should be combined with kinetic modeling and simulation in order to correlate theoretical results with experimental trends or even predict the catalytic activity and selectivity of novel materials.

**Figure 8:**
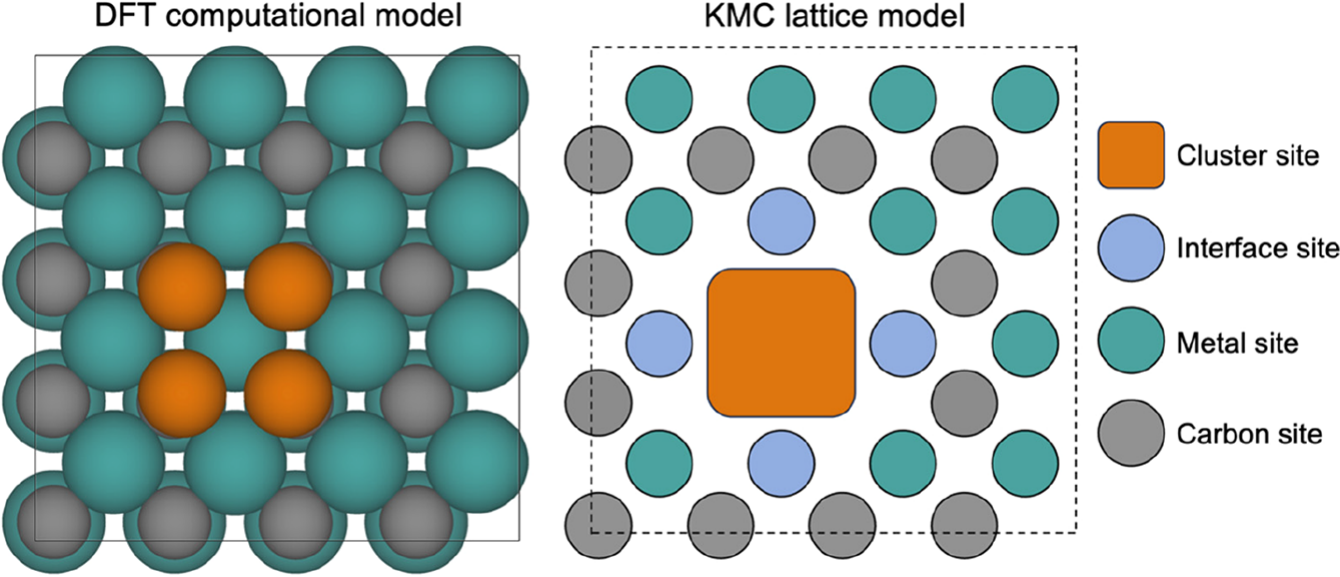
Example of a computational model for a supported cluster on an *fcc* TMC(001) facet and a possible lattice model for KMC simulations, showing a variety of site types.

When simulating the temporal evolution at the TM/TMC surface, one needs to consider three main catalytic regions: the supported TM cluster, the TMC support, and the TM–TMC interface, implying that the kinetic model must be able to deal with complex lattices of active sites (Figure 8). Moreover, the binding of some adsorbed intermediates on TM/TMCs can be very strong,^51^ leading to a significant coverage, and diffusion processes are slow in general,^14^ implying that the mean-field approximation is likely to break down. Therefore, kinetic Monte Carlo (KMC) simulations,^53,54^ which do not rely on the mean-field approximation, are especially suited to model the reactivity of TM/TMCs. Indeed, KMC simulations are spatially resolved and capture, among other things, spatial correlations and ordering arising from adsorbate lateral interactions and changes in the activation energies of elementary events due to lateral interactions with neighboring spectator species.

The first KMC study on TM/TMCs explored the reaction mechanism of the WGS (CO + H_2_O → CO_2_ + H_2_) on Au/MoC.^13^ The study was inspired by experimental results, which showed that the catalytic activity for that reaction strongly depends on the Au coverage, with the maximum activity corresponding to a coverage of ∼0.15 ML,^42^ approximately 7 times higher compared to clean MoC. Preliminary DFT calculations on Au_4_/MoC suggested that Au clusters promote the direct CO oxidation (i.e., the calculated energy barrier for CO + O → CO_2_ lowers from 2.22 eV in MoC to only 0.44 eV in Au_4_/MoC). However, first-principles based KMC simulations on a lattice model corresponding to an Au coverage of ∼0.15 ML and including several types of adsorption sites provided strong evidence for a cooperative effect between the different regions of the catalyst. While MoC was shown to be responsible for water dissociation, the Au–MoC interface promotes COOH formation, a crucial intermediate for the production of CO_2_ (i.e., CO + OH → COOH → CO_2_ + H), and speeds up product desorption. Moreover, KMC simulations on different lattice models corresponding to a coverage of ∼0.25 and 0 ML (i.e., clean MoC) predicted a catalytic activity ∼4 and ∼8 times lower, respectively, in remarkable agreement with experimental results. This study confirmed the active role of the TMC support, which not only activates the supported metal particles by polarization of the electron density but is also responsible for some reaction steps, leading to a bifunctional catalyst.

First-principles based KMC simulations were also employed very recently to study the reverse WGS (RWGS) reaction on Ni/TiC.^14^ Experiments had shown that the catalytic activity for the RWGS on TiC can be boosted by dispersing small Ni particles, resulting in an increase of 2 orders of magnitude. DFT-derived free energy diagrams suggest that the catalytic activity decreases in the order Ni > interface > TiC, with the *direct* CO_2_ dissociation pathway being dominant compared to the *assisted* pathway (i.e., CO_2_ + H → COOH → CO + OH). These conclusions, however, did not agree with KMC simulation results, which suggest that the *assisted* pathway is dominant and 97% of the product CO molecules are produced on TiC. This discrepancy arises from the coverage effects, as both the Ni clusters and the interface are poisoned by OH species, but H spillover from the Ni clusters to TiC promotes the formation of COOH on TiC and prevents its partial oxidation by O. Specifically, the coverage of the O* species decreases from ∼46% (clean TiC) to ∼13% (TiC region of Ni/TiC), while that of H* increases from ∼1% to ∼35%. The KMC simulations reproduce the experimentally observed 2 orders of magnitude increase in activity on Ni/TiC compared to TiC, and, crucially, the predicted turnover frequencies (TOFs) are in quantitative agreement with experiments. This work elucidates the limitations of free energy diagrams to understand the catalytic activity of TM/TMCs and other complex catalysts in general, as they do not easily account for the effect of surface coverage, which can play a critical role, and highlights the importance of KMC simulations as a fundamental tool to delve deeper into the inner workings of complex catalysts.

## Key Factors Defining Activity, Selectivity, and Stability

The catalytic performance of TM/TMCs depends on multiple factors. One of the most important parameters is the size of the supported clusters. Experimental studies have revealed that the catalytic activity improves substantially when the size of these particles is very small (<0.6 nm),^28^ and DFT calculations have explained this higher reactivity on the basis of a high degree of charge polarization.^12^ In general, small clusters have a planar geometry (i.e., two-dimensional), as pointed out by STM studies^29^ and DFT calculations.^50^ Clusters with a distribution of small sizes can be obtained only when the coverage is low. For admetal coverages above 0.15–0.2 ML, larger three-dimensional particles start to grow, usually leading to a decrease in reactivity, as is the case for instance in the WGS reaction on Au/MoC, where a maximum in the production of H_2_ and CO_2_ is observed at a Au coverage of 0.15 ML; after this point there is a gradual decrease in activity.^42^

Despite the existing understanding of the impact of size on the catalytic activity and stability, there remains a gap in our understanding regarding the influence of having multiple isomers among the supported clusters. As discussed by Alexandrova et al.^55^ in the context of oxide-supported clusters, the catalytic interface should be viewed as an evolving statistical ensemble of many structures rather than the single most thermodynamically stable one, and the kinetics of the catalytic process may be governed by the presence of more active yet less prevalent metastable isomers. Consequently, theoretical calculations on different isomers are highly desirable to assess to which extent one should worry about the fluxionality of the supported clusters.

In the limit of the smallest size possible, TMCs have also been shown to provide new opportunities for the synthesis of stable SACs (TM_1_/TMC). For instance, Pt could be atomically dispersed on MoC, and the resulting Pt_1_/MoC SAC was highly active and selective for APRM and selective hydrogenation of substituted nitroarenes.^44^ The stability of TMC-supported SACs can be improved with surface metal vacancies in the TMC support, which favors the supported TM single atoms to occupy the metal vacancy and prevents their aggregation. This was recently shown by Ma et al.,^56^ who designed a Pd_1_/MoC catalyst with high activity and excellent selectivity for liquid-phase hydrogenation of substituted nitroaromatics and gas-phase hydrogenation of CO_2_ to CO that could endure temperatures up to 400 °C without any observable aggregation of Pd atoms.

Another important parameter is the metal/C ratio of the carbide support. In general, a decrease in metal/C ratio reduces the reactivity as a consequence of electronic (a raise in the positive charge on the metal) and structural effects (less metal centers exposed).^41^ Theoretical calculations indicate that CO_2_ adsorbs molecularly on TiC(001) and MoC(001),^57,58^ and the cleavage of the C–O bond occurs only after hydrogenation of CO_2_ forming a COOH intermediate.^27^ On the contrary, CO_2_ dissociates rather easily on Mo_2_C, and the dissociation of the second C–O bond requires overcoming only a small activation barrier.^57^ The resulting C atoms are then hydrogenated to produce methane. Thus, TMCs with 1:1 stoichiometry are more resistant against O* poisoning^29^ and, as mentioned earlier, MoC was shown to be a much more stable catalyst support for Au, Cu, and Pt particles compared to Mo_2_C,^39,41,42^ as it does not deactivate by the formation of an oxycarbide. Experimentally, a range of TM_*x*_C compositions can be fabricated by varying the metal precursor/C precursor ratio during its synthesis,^59^ but note that some carbides such as group IV TMCs (i.e., Ti, Zr and Hf) only have a stable phase corresponding to a 1:1 stoichiometry (Figure 1), so the range of possible metal/C ratio values for these carbides would be limited by the maximum number of C vacancies that can be created.

Equally significant is the crystalline phase of the TMC support. Hexagonal TMCs have been shown to bind adsorbates much more strongly than cubic TMCs due to a shift in the *d*-band center toward higher energies.^51^ In line with what has been discussed above for the metal/C ratio, this implies that hexagonal TMCs are more prone to be deactivated due to oxycarbide formation, making them less attractive for catalytic applications. Another obvious factor is the nature of the metal atoms in the TMC and the cluster. Regarding the nature of the TMC, their affinity for O* species increases down a group (e.g., VC < NbC < TaC), and DFT calculations indicate that metal particles supported on group IV and V carbides are significantly more resistant against fragmentation and more stable in the presence of adsorbates (Figure 6).^52^ Finally, most metal clusters supported on group IV TMCs can break CH_4_ and CO_2_ with very low energy barriers, while for group V and IV TMCs this only occurs for the case of TaC and MoC supported clusters, with the energy barriers for the dissociation of CH_4_ and CO_2_ decreasing when going down a group (Figure 7). The nature of the supported cluster is less important in terms of stability as the stability is mainly determined by the carbide support. The catalytic activity and selectivity of supported clusters, however, will greatly depend on the metal cluster used. For instance, the selectivity of the CO_2_ hydrogenation on Mo_2_C toward methanol, methane, and alkanes can be improved by depositing Cu, Ni, and Co clusters, respectively.^16^ Also, supported Pt clusters always exhibit very low energy barrier for the dissociation of CH_4_ and CO_2_, regardless of the TMC support.^52^

On a final note, the amount of surface C vacancies can also affect the stability and reactivity of these materials. Note that TMCs are almost never stoichiometric in practice, mainly due to slow diffusion rates for C penetration into the metal lattices during the synthesis. DFT calculations indicate that surface C vacancies increase the binding strength of the supported clusters as a result of a higher charge transfer from the TMC support to the metal cluster, making them more stable.^50^ In addition, C vacancies can significantly modify the catalytic activity of the TMC due to the creation of new types of active sites and more active metal centers. For instance, Pajares et al. showed that the presence of C vacancies boosts the activity of the RWGS reaction on VC catalysts by performing the reaction on two VC samples, one mainly stoichiometric and another being C-deficient.^9^ DFT calculations confirmed that C vacancies in the C-deficient sample are responsible for the observed catalytic behavior, allowing reactants to adsorb more strongly and lowering the energy barrier for both H_2_ and CO_2_ dissociation steps.

## Summary and Future Directions

The use of TMCs as catalytically active supports for metal particles opens up a plethora of opportunities for catalyst design, as the catalytic behavior of these TM/TMC systems can be tuned by choosing the appropriate combination of metal cluster and carbide support, optimizing the adsorption strength of key species.^51^ Currently, only very few TM/TMCs have been tested experimentally, all of them involving either TiC or Mo_*x*_C as a support,^41^ which calls for further experimental studies on other carbides. High-throughput screening based on DFT is key to shed some light on the stability and reactivity of these systems and determine which combinations may best catalyze which chemical reactions by the identification of effective descriptors,^50,52^ while deeper analysis into their inner workings require KMC simulations accounting for both TM and TMC active sites.^13,14^ Variations in the cluster-carbide combination, size and shape of the nanoclusters, metal/C ratio, and the number of surface C vacancies can offer significant opportunities to tune the stabilities of key intermediates and thus the overall catalytic activity and selectivity. A close interaction between theoretical modeling and experimental studies on well-defined systems is of great importance to understand the interplay of the different active sites in these multifunctional catalysts and optimize them for target processes.

Apart from their use as supports in thermocatalysis, TMCs have also attracted attention as electrocatalyst supports since they are chemically stable in acidic media, resistant to poisoning, and possess high electronic conductivity. For instance, TMCs have been shown to promote the stability and intrinsic activity of supported Pt particles for the hydrogen evolution reaction (HER) and the oxygen reduction reaction (ORR).^60^

**Figure 9:**
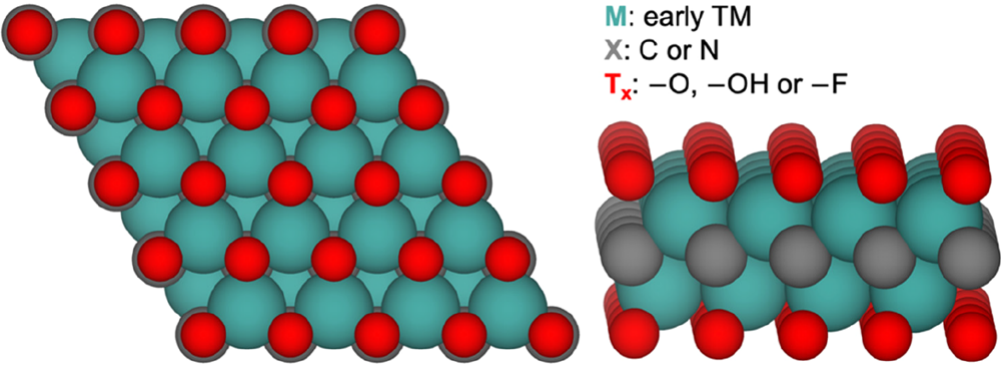
Top and side view of a M_2_XT_*x*_ MXene.

Finally, MXenes, a recently discovered family of two-dimensional (2D) transition metal carbides or nitrides,^61^ with the formula M_*n*+1_X_*n*_T_*x*_ (Figure 9, where M is an early transition metal, T_*x*_ is a surface termination group, X = C and/or N), have garnered increasing attention for nearly a decade in the context of various applications, among which is catalysis.^62^ The main advantages of MXenes compared to 2D TMCs are their greater specific surface area, better stability under oxidative environments,^63^ and the fact that they have only one well-determined metal-terminated plane, which makes them ideal to study computationally, without having to invoke any major assumptions or simplifications. Their versatile composition and structure, stability under conditions of interest in heterogeneous catalysis, large surface area, and abundant anchoring sites (such as surface defects) make them attractive supports for single atoms or small particles.^64^

To date, a series of single heteroatoms have been supported or incorporated into the lattices of MXenes. For instance, Ti_3–*y*_C_2_T_*x*_ MXene nanosheets characterized by abundant Ti-deficit vacancy defects were used as supports for Pt single atoms, which form strong metal–carbon bonds with the support and are therefore stabilized onto the sites previously occupied by Ti.^65^ The resulting Pt-based SAC (Pt_1_/Ti_3–*y*_C_2_T_*x*_) is very active toward the functionalization of CO_2_ via the formation of amines, as a C_1_ source in organic synthesis. DFT calculations revealed that such single Pt atoms feature partial positive charges and atomic dispersion, which significantly decrease the adsorption energy and activation energy of silane, CO_2_, and aniline, thereby boosting the catalytic performance. In another recent joint experimental and computational study, Zhou and co-workers showed that silica-supported Cu_1_/Mo_2_CT_*x*_ MXene catalyst hydrogenates CO_2_ to methanol with >50% selectivity and higher intrinsic methanol formation rate per mass than the reference Cu catalysts, while not deactivating with time on stream.^66^ DFT calculations elucidated the critical role of the interface between Cu and the partially defunctionalized Mo_2_CT_*x*_, which stabilizes key reaction intermediates. Still though, a hurdle to overcome in the path toward the practical use of MXenes is their synthesis in industrial quantities, although several steps in this direction have recently been taken.^67^ The knowledge acquired by the study of MXenes can also be used to guide the rational design and fabrication of more economical 3D TMC-based catalysts.
